# [Expression of Concern] EETs and CYP2J2 inhibit TNF-α-induced apoptosis in pulmonary artery endothelial cells and TGF-β1-induced migration in pulmonary artery smooth muscle cells

**DOI:** 10.3892/ijmm.2026.6012

**Published:** 2026-10-12

**Authors:** Wenjing Feng, Xizhen Xu, Gang Zhao, Geng Li, Tiantian Liu, Junjie Zhao, Ruolan Dong, Dao Wen Wang, Ling Tu

Int J Mol Med 32: 685-693, 2013; DOI: 10.3892/ijmm.2013.1435

Following the publication of the above paper, it has been drawn to the Editor's attention by a concerned reader that, for the cell migration assay experiments shown in Fig. 6A on p. 691, the 'Control' and 'TGF-β1 + 14,15-EET' data panels contained an overlapping section of data, such that these data, which were intended to show the results from differently performed experiments, appeared to have been derived from the same original source.

The authors have been contacted by the Editorial Office to offer an explanation for the apparently incorrect assembly of data in Fig. 6, and we are awaiting their response. Owing to the fact that the Editorial Office has been made aware of potential issues surrounding the scientific integrity of this paper, we are issuing an Expression of Concern to notify readers of this potential problem while the Editorial Office conitnues to investigate this matter further.

